# Improved survival rates in patients with H1N1 acute respiratory failure in Korea between 2009 and 2016

**DOI:** 10.1371/journal.pone.0223323

**Published:** 2019-10-03

**Authors:** Hayoung Choi, Ui Won Ko, Hyun Lee, Sang-Bum Hong, Chi Ryang Chung

**Affiliations:** 1 Division of Pulmonary, Allergy, and Critical Care Medicine, Department of Internal Medicine, Hallym University Kangnam Sacred Heart Hospital, Seoul, Korea; 2 Department of Critical Care Medicine, Samsung Medical Center, Sungkyunkwan University School of Medicine, Seoul, Korea; 3 Division of Pulmonary Medicine and Allergy, Department of Internal Medicine, Hanyang University College of Medicine, Seoul, Korea; 4 Division of Pulmonary and Critical Care Medicine, Department of Medicine, Asan Medical Center, University of Ulsan College of Medicine, Seoul, Korea; 5 Department of Medicine, Samsung Medical Center, Sungkyunkwan University School of Medicine, Seoul, Korea; Harvard School of Public Health, UNITED STATES

## Abstract

There was a pandemic of influenza A (H1N1) in 2009; in Korea, there was also an H1N1 epidemic in 2016. We aim to investigate whether survival had improved in the setting of recent advances in intensive care unit (ICU) management. We conducted a retrospective analysis of acute respiratory failure patients with H1N1 influenza pneumonia in 2016 and 2009 respectively at two tertiary referral hospitals in Korea. A total of 28 patients were treated in 2016, and 34 in 2009. There was no significant difference in SOFA scores on ICU admission day. In-hospital mortality was significantly lower in patients of 2016 compared to those of 2009 (18% vs. 44% *P* = 0.028). By multivariable analyses, the treatment year 2016 was associated with a greater likelihood of survival. Compared to the patients treated in 2009, those treated in 2016 were one seventh as likely to die after adjusting for other clinical variables (hazard ratio for mortality, 0.15; 95% confidence interval. 0.03–0.63, *P* = 0.010). Improved survival in patients who underwent extracorporeal membrane oxygenation treatment (in-hospital mortality, 17% vs. 60%, *P* = 0.242) and decreased tidal volumes during mechanical ventilation (median 5.4 mL/kg vs. median 9.2 mL/kg, *P* = 0.018) were observed in 2016 compared to 2009. Treatment outcomes for patients with H1N1 acute respiratory failure improved from 2009 to 2016 in two tertiary referral centers in South Korea.

## Introduction

The World Health Organization declared a global influenza pandemic in 2009 when the influenza A (H1N1) pdm09 virus spread rapidly worldwide [1]. In Korea, 740,835 patients were confirmed to have pandemic H1N1 in 2009 and 225 died [2,3]. During the pandemic period, some patients were hospitalized in intensive care units (ICU) because of influenza-associated pneumonia and acute respiratory distress syndrome (ARDS) [4]. The development of ARDS led to a tremendous increase in the mortality rate, ranging from 17.3 to 41.4% among critically ill patients with H1N1 infection [5–7].

Multiple aspects of ICU patient management have improved in recent years, including the more widespread application of lower tidal volume ventilation [8], restrictive blood transfusion [9], reduced sedative use [10], early mobilization, and more appropriate intervention in patients with sepsis [11,12]. Also, some lessons were learned while managing patients with 2009 H1N1-related critical illnesses, and extracorporeal membrane oxygenation (ECMO) therapy has been applied for the most severe ARDS associated with H1N1 infection [13,14].

In Korea, there was also the 2016 influenza season dominated by influenza A (H1N1) pdm09 virus. We hypothesized that survival had improved in patients with a viral infection between 2009 and 2016 because of advances in ICU management. We aimed to investigate whether treatment outcomes in 2016 had improved compared to those in 2009 in patients with H1N1 acute respiratory failure as well as which treatment modalities were associated with favorable treatment outcomes.

## Materials and methods

### Study population

We enrolled patients aged ≥ 15 years, who were diagnosed with H1N1 acute respiratory failure and admitted to the ICU at Samsung Medical Center (a 1,979-bed referral hospital in Seoul, Korea) or Asan Medical Center (a 2,704-bed referral hospital in Seoul, Korea). We defined H1N1 acute respiratory failure as an H1N1 infection requiring mechanical ventilator (MV) support. Data were collected from all consecutive patients from November 2009 to February 2010 and from November 2015 to March 2016, which were analyzed retrospectively. Patient data during the 2009 pandemic period were extracted from a multicenter cohort that investigated adult patients with confirmed H1N1-related critical illnesses who admitted to ICUs at 28 Korean tertiary or referral hospitals [15]. Among 57 critically ill patients of Samsung Medical Center and Asan Medical Center in the database, this study included 34 patients with H1N1 acute respiratory failure. This retrospective study was approved by the institutional review board (IRB) of Samsung Medical Center (IRB application no. 2016-07-137) and Asan Medical Center (IRB application no. 2018–0224). Patient information was anonymized and de-identified before analysis; therefore, requirements for informed consent were waived.

### Data collection

Patient medical records were reviewed and clinical data were extracted, including demographic characteristics, body mass index (BMI), smoking history, diagnostic method, Sequential Organ Failure Assessment (SOFA) score, nosocomial infection, and comorbidities (hypertension, diabetes mellitus, chronic obstructive pulmonary disease [COPD], chronic kidney disease, chronic liver disease, current or previous solid organ malignancy). We used SOFA score to assess severity of patients because Acute Physiology and Chronic Health Evaluation (APACHE) II score were not appropriate in patients transferred from outside hospitals on extracorporeal membrane oxygenation (ECMO) support. SOFA score was assessed upon admission. Data about treatment in the ICU were also extracted, including antiviral agents, antibiotics, corticosteroids, MV settings (fraction of inspired oxygen [FiO_2_], positive end-expiratory pressure [PEEP], inspiratory pressure, and tidal volume for predicted body weight), neuromuscular blocking agents, prone position, nitric oxide, continuous renal replacement therapy (CRRT), and ECMO. The predicted body weight was calculated using the following equations: predicted body weight (kilograms) for men = 50 + (0.91 * [height {centimeters}]– 152.4]); predicted body weight (kilograms) for women = 45.5 + (0.91 * [height {centimeters}– 152.4]) [8]. In addition, we documented outcomes of patients with H1N1 acute respiratory failure, including the length of ICU and hospital stay and in-hospital mortality.

### Statistical analysis

Data are presented as the median and interquartile range (IQR) for continuous variables and as frequency (percentage) for categorical variables. Data were compared by the Mann-Whitney *U* test for continuous variables and by the Pearson’s *chi*-square test or Fisher’s exact test for categorical variables. A Cox proportional hazard model was used to determine whether treatment year associated with survival. We further evaluated which factors associated with favorable treatment outcomes. The initial clinical variables entered into the model included age, sex, BMI, year, diabetes mellitus, solid organ malignancy, neuromuscular blockers, prone position, nitric oxide, ECMO, and CRRT in acute kidney injury. All tests were two-sided, and p < 0.05 was considered significant. Data were analyzed using IBM SPSS Statistics for Windows, version 23.0 (Armonk, NY, USA).

## Results

### Patient characteristics

In 2009, 34 patients diagnosed with H1N1 acute respiratory failure; 18 patients at Samsung Medical Center and 16 at Asan Medical Center. In 2016, there were 28 patients diagnosed; 16 at Samsung Medical Center and 12 at Asan Medical Center. Some clinical data for patients treated in 2009 were included in a published article [15,16]; data on patients in 2016 have not been previously reported.

Baseline characteristics are summarized in Table 1. The median age of all patients was 62 years (IQR, 53–69 years) and 63% of all patients were male. Patients in 2016 were older (67 versus 60 years old, *P* = 0.109) and had higher BMI (23.1 versus 21.4 kg/m^2^, *P* = 0.013) than those in 2009. There was no significant difference in SOFA scores on ICU admission day (9 versus 10, *P* = 0.208) between patients diagnosed in 2009 and 2016. Solid organ malignancies were less frequently observed in patients in 2016 than in those in 2009 (18% versus 41%, *P* = 0.047).

**Table 1:** Baseline characteristics of patients with H1N1 acute respiratory failure.

	2016 (n = 28) number (%), median (IQR)	2009 (n = 34) number (%), median (IQR)	*P-value*
Age, years	67 (57–71)	60 (49–67)	0.109
Male sex	15 (54)	24 (71)	0.167
Body mass index, kg/m^2^	23.1 (21.3–26.9)	21.4 (19.1–24.3)	0.013
Smoking	11 (39)	17 (50)	0.399
Diagnostic methods			
RT-PCR	25 (89)	34 (100)	0.087
SOFA score	9 (5–12)	10 (8–12)	0.208
Hospital-acquired H1N1 infection	5 (18)	13 (38)	0.079
Comorbidities			
Hypertension	11 (39)	12 (35)	0.746
Diabetes mellitus	11 (39)	9 (27)	0.283
Chronic obstructive pulmonary disease	3 (11)	4 (12)	1.0
Chronic kidney disease without hemodialysis	0	2 (6)	0.497
Chronic kidney disease on hemodialysis	5 (18)	4 (12)	0.719
Chronic liver disease	4 (14)	3 (9)	0.691
Current or previous solid organ malignancy	5 (18)	14 (41)	0.047

IQR, interquartile range; BMI, body mass index; RT-PCR, reverse transcription polymerase chain reaction; SOFA, Sequential Organ Failure Assessment.

### Treatment modalities and outcomes

Treatment modalities and outcomes are summarized in Table 2. Antiviral agents were used in all patients in both years. Peramivir was more frequently used in patients diagnosed in 2016 than in those diagnosed in 2009 (93% versus 21%, *P* < 0.001). MV settings including FiO_2_, PEEP, inspiratory pressure, and tidal volume for predicted body weight were not significantly different between the two groups of patients. Six (21%) patients in 2016 and 15 (44%) in 2009 received CRRT. Among six patients in 2016, four had chronic kidney disease on hemodialysis and two had acute kidney injury, without underlying chronic kidney disease; among 15 patients in 2009, four patients had chronic kidney disease on hemodialysis, two had chronic kidney disease without hemodialysis, and nine had acute kidney injury without underlying chronic kidney disease. There was no significant difference in the use of adjunctive treatment modalities including neuromuscular blocking agents, prone positioning, and nitric oxide between the two years. ECMO was used for 21% (n = 6) of patients in 2016 and for 15% (n = 5) of those in 2009 (*P* = 0.523). Four of the six patients treated with ECMO in 2016 had been transferred from outside hospitals on ECMO support.

**Table 2:** Treatment modalities and outcomes of patients with H1N1 acute respiratory failure.

	2016 (n = 28) number (%), median (IQR)	2009 (n = 34) number (%), median (IQR)	*P-value*
Antiviral treatment			< 0.001
Oseltamivir	2 (7)	17 (50)	
Peramivir	26 (93)	7 (21)	
Oseltamivir, amantadine and ribavirin	0	10 (29)	
Antibiotics	28 (100)	34 (100)	NA
Corticosteroids	16 (57)	25 (74)	0.175
Mechanical ventilation			
FiO2	0.5 (0.4–0.6)	0.55 (0.4–0.6)	0.507
PEEP, cmH_2_O	8 (5–10)	9 (6–10)	0.193
Inspiratory pressure, cmH_2_O	14 (12–16)	12 (9–16)	0.451
Tidal volume, mL/kg	6.4 (5.2–8.2)	7.3 (5.9–9.2)	0.103
Neuromuscular blocking agent	18 (64)	25 (74)	0.432
Prone positioning	4 (14)	5 (15)	1.0
Nitric oxide	3 (11)	7 (21)	0.490
CRRT	6 (21)^a^	15 (44)^b^	0.060
CRRT in acute kidney injury	2 (7)	9 (27)	0.092
ECMO	6 (21)	5 (15)	0.523
Length of MV, days	7 (4–19)	6 (3–17)	0.571
Length of ICU stay, days	10 (7–28)	8 (5–14)	0.210
Length of hospital stay, days	20 (11–57)	20 (11–36)	0.508
In-hospital mortality	5 (18)	15 (44)	0.028

IQR, interquartile range; NA, not applicable; FiO2, fraction of inspired oxygen; PEEP, positive end-expiratory pressure; CRRT, continuous renal replacement therapy; ECMO, extracorporeal membrane oxygenation; MV, mechanical ventilation; ICU, intensive care unit.

^a^ Four patients had chronic kidney disease on hemodialysis, and two had acute kidney injury, without underlying chronic kidney disease

^b^ Four patients had chronic kidney disease on hemodialysis, two had chronic kidney disease without hemodialysis, and nine had acute kidney injury, without underlying chronic kidney disease.

**Fig 1:**
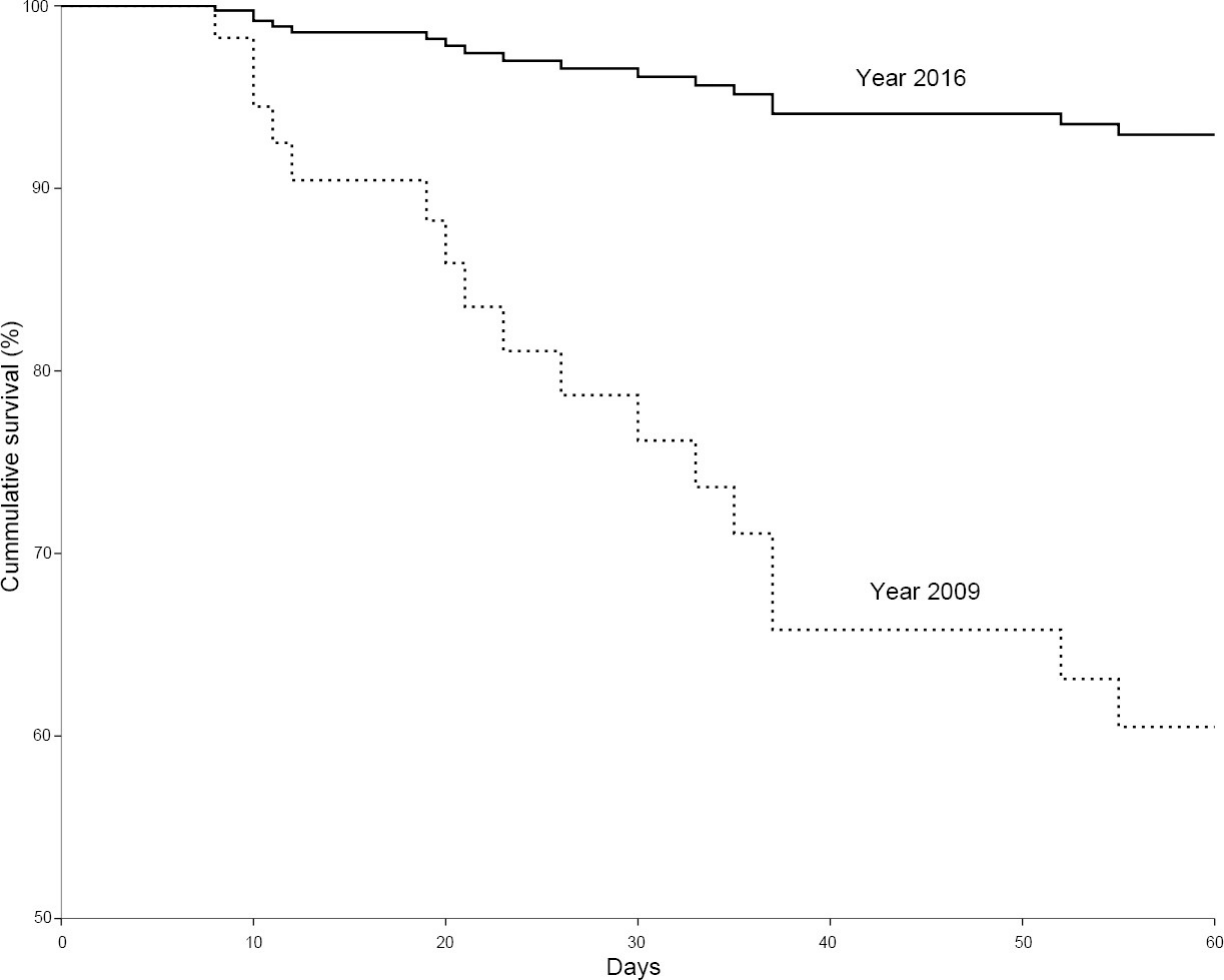
Cox proportional hazards model cumulative survival curve for patients with H1N1 acute respiratory failure each year (the solid line represents patients treated in 2016; the dotted line represents those treated in 2009).

In-hospital mortality was significantly lower in patients treated in 2016 compared to those treated in 2009 (18% versus 44%, *P* = 0.028). There was no significant difference in length of stays in the ICU and hospital between the two years (Table 2). As shown in Fig 1, the cumulative survival rate was significantly higher in acute respiratory failure patients with H1N1 in 2016 compared to those in 2009. Patients in 2016 were one seventh as likely to die after adjusting for other clinical variables (hazard ratio [HR], 0.15; 95% confidence interval [CI], 0.03–0.63, *P* = 0.010). Additionally, age (HR, 1.05; 95% CI, 1.01–1.10, *P* = 0.028), current or previous solid organ malignancy (HR, 0.17; 95% CI, 0.04–0.81, *P* = 0.025), and prone positioning (HR, 7.52; 95% CI, 1.37–41.40, *P* = 0.020) were significantly associated with mortality (Table 3).

**Table 3:** Results of univariable and multivariable Cox proportional hazard model of mortality in patients with H1N1 acute respiratory failure.

	Univariable analysis		Multivariable analysis	
	HR	*P-value*	HR	*P-value*
Age	1.02 (0.99–1.06)	0.217	1.05 (1.01–1.10)	0.028
Male sex	1.16 (0.46–2.91)	0.751	1.41 (0.36–5.53)	0.620
BMI	1.01 (0.94–1.09)	0.765	1.07 (0.96–1.20)	0.218
Year 2016	0.33 (0.12–0.91)	0.331	0.15 (0.03–0.63)	0.010
Diabetes mellitus	0.85 (0.33–2.21)	0.737	1.22 (0.41–3.61)	0.726
Current or previous solid organ malignancy	1.16 (0.46–2.90)	0.756	0.17 (0.04–0.81)	0.025
Neuromuscular blocking agent	1.96 (0.66–5.88)	0.227	3.34 (0.83–13.39)	0.089
Prone positioning	3.45 (1.32–9.02)	0.012	7.52 (1.37–41.40)	0.020
Nitric oxide	2.69 (1.03–7.01)	0.044	0.16 (0.02–1.55)	0.112
ECMO	1.18 (0.39–3.53)	0.769	1.27 (0.19–8.38)	0.804
CRRT in acute kidney injury	3.71 (1.51–9.13)	0.004	4.15 (0.83–20.64)	0.082

A Cox proportional hazard model was used to determine whether treatment year was associated with in-hospital mortality. The initial clinical variables entered in the model included age, sex, body mass index, year, diabetes mellitus, current or previous solid organ malignancy, neuromuscular blockers, prone positioning, nitric oxide, ECMO, and CRRT in acute kidney injury.

OR, odds ratio; HR, hazard ratio; CI, confidence interval; BMI, body mass index; ECMO, extracorporeal membrane oxygenation; CRRT, continuous renal replacement therapy.

### ECMO treatment

Characteristics, treatment modalities, and outcomes of H1N1 patients who received ECMO treatment are summarized in Table 4. Patients in 2016 (n = 6) tended to be younger (55 versus 60 years, *P* = 0.522) and had higher BMIs (22.1 versus 19.4 kg/m^2^, *P* = 0.068) than did those in 2009 (n = 5), although neither of these differences were statistically significant. On admission day, there was no significant difference in SOFA scores (14 versus 13, *P* = 0.310) between the two years. At the time of initiating ECMO treatment, tidal volumes for predicted body weight (5.4 versus 9.2 mL/kg, *P* = 0.018) and FiO_2_ (0.5 versus 0.9, *P* = 0.009) were significantly lower in 2016 than in 2009. There was no significant difference in the length of hospital and ICU stay between the two years. In-hospital mortality (17 versus 60%, *P* = 0.242) was lower in 2016 than in 2009.

**Table 4:** Characteristics, treatment modalities, and outcomes of patients with H1N1 acute respiratory failure who received ECMO treatment.

	Total (n = 11) number (%), median (IQR)	2016 (n = 6) number (%), median (IQR)	2009 (n = 5) number (%), median (IQR)	*P-*value
Age, years	58 (41–60)	55 (39–59)	60 (33–71)	0.522
Male sex	5 (46)	3 (50)	2 (40)	1.0
BMI, kg/m^2^	21.5 (19.4–23.3)	22.1 (21.3–28.2)	19.4 (14.6–22.7)	0.068
Smoking	3 (27)	2 (33)	1 (20)	1.0
SOFA score	14 (8–16)	14 (10–17)	13 (6–15)	0.310
Nosocomial infection	2 (18)	1 (17)	1 (20)	1.0
Antibiotics	11 (100)	6 (100)	5 (100)	1.0
Corticosteroid	5 (46)	2 (33)	3 (60)	0.567
Mechanical ventilation				
FiO_2_	0.6 (0.4–1.0)	0.5 (0.4–0.6)	0.9 (0.8–1.0)	0.009
PEEP, cmH_2_O	10 (8–14)	10 (7–16)	10 (8–13)	0.852
Inspiratory pressure, cmH_2_O	11 (10–13)	11 (10–12)	12 (NA)	0.601
Tidal volume, mL/kg	6.8 (5.0–9.2)	5.4 (2.7–7.1)	9.2 (7.5–12.3)	0.018
Neuromuscular blocking agent	10 (91)	5 (83)	5 (100)	1.0
Prone positioning	4 (36)	2 (33)	2 (40)	1.0
Nitric oxide	6 (55)	2 (33)	4 (80)	0.242
CRRT	5 (46)	1 (17)	4 (80)	0.080
Length of MV, days	12 (6–20)	9 (6–19)	12 (4–21)	0.926
Length of ICU stay, days	14 (8–28)	20 (8–39)	7 (14–21)	0.584
Length of hospital stay, days	20 (11–50)	34 (11–53)	20 (12–26)	0.584
In-hospital mortality	4 (36)	1 (17)	3 (60)	0.242

IQR, interquartile range; ECMO, extracorporeal membrane oxygenation; BMI, body mass index; APACHE, Acute Physiology and Chronic Health Evaluation; SOFA, Sequential Organ Failure Assessment; FiO_2_, fraction of inspired oxygen; PEEP, positive end-expiratory pressure; CRRT, continuous renal replacement therapy; MV, mechanical ventilation; ICU, intensive care unit; NA, not applicable.

## Discussion

In this study, we investigated whether survival had improved in patients with H1N1 acute respiratory failure because of advances in ICU management and sought to identify which treatment modalities had been used during an epidemic in 2016 and a pandemic in 2009. In-hospital mortality was 18% among patients in 2016 while it was 44% in 2009. The improved survival was associated with treatment year though there was no association between specific treatment modalities and improved treatment outcomes.

The mortality in this study (18% in 2016 and 44% in 2009) was comparable with the 17.3–41.4% mortality rate reported by previous studies of critically ill patients with H1N1 infection [5,6]. This improved survival might be related to advances in ICU management. Regarding MV parameters, tidal volumes were lower in H1N1 patients of the 2016 influenza season compared to those of the 2009 influenza, despite no statistical significance. This may imply that intensivists have more tendency to recognize ARDS in a timely manner and adhere to low tidal volumes, which may influence improved survival in this study [17]. In this study, prone positioning seemed to be associated with increased mortality in patients with H1N1 acute respiratory failure. However, it might have resulted from the phenomenon that prone positioning had been implemented to patients suffering from severe hypoxia, who were in the worst clinical settings. In addition, prone positioning showed decreased 28-day and 90-day mortality and increased ventilator-free days in patients with severe ARDS [18]. Thus, we should be cautious in interpreting the results.

After the H1N1 pandemic, several institutions reported observational data suggesting ECMO increased survival in severe H1N1 pneumonia [13,19]. The reports were consistent with our finding that the proportion of patients receiving ECMO treatment increased from 15% (in 2009) to 21% (in 2016). In addition, in-hospital mortality of those who received ECMO was lower in 2016 (17%) than in 2009 (60%), although there was no significant association between improved survival and ECMO in a Cox proportional hazard model. In H1N1 ARDS, high tidal volumes (> 9 mL/kg for predicted body weight) and time of deoxygenation (Pao_2_ < 70 mm Hg) longer than 6 hours were associated with high mortality [20,21]. In this study, it was notable that tidal volumes for predicted body weight were significantly lower in patients receiving ECMO in 2016 than in those in 2009, which might explain the improved survival. Thus, prompt initiation of ECMO treatment should be considered when refractory hypoxia is not relieved by conventional treatment in severe H1N1 pneumonia.

In this study, CRRT was more frequently used with acute kidney injury in 2009 than in 2016. However, CRRT in acute kidney injury was not associated with decreased in-hospital mortality. Our findings were aligned with previous studies, which reported early renal replacement therapy initiation strategy was not associated with severe acute kidney injury and septic shock or ARDS [22]. Also, other observational study revealed that long-term risk of major cardiovascular events in ICU survivors was more common in those who had received renal replacement therapy than in those who had not received renal replacement therapy [23]. However, since the study population who received CRRT in our study was small and there are no relevant studies which evaluated the role of CRRT in H1N1 patients with AKI, further studies are needed to confirm the role of CRRT in the treatment outcomes in this population.

There is always a chance of other endemic or pandemic viral infections in the future. The 2015 outbreak of Middle East Respiratory Syndrome coronavirus in Korea is one such example [24]. However, unfortunately treatment outcomes in patients with severe viral pneumonia during a pandemic period are reportedly poor without definitive treatment modalities [5–7]. From this perspective, our study provides valuable data suggesting that advances in routine ICU management may improve treatment outcomes in such viral infections. Thus, investment and effort to improve ICU care are crucial to improving treatment outcomes in patients with a severe viral infection.

Our study has several limitations. First, it was a retrospective study conducted at two referral centers in Korea. Second, the sample size was too small. Patients in 2016 were more likely to receive lower tidal volumes than those in 2009 and treatment outcomes in patients who received ECMO in 2016 were better than those in 2009. However, these factors were not statistically significant probably due to the small sample size. Though it was not proven in this study, prompt ECMO and lung-protective ventilation may have led to advances in ICU management that have resulted in improved survival. Third, its small sample size made our study prone to selection bias. Multivariable analysis might not be enough to remove selection bias. Thus, future studies with larger study populations are needed to investigate specific treatment modalities to improve survival in acute respiratory failure patients with H1N1 pneumonia.

## Conclusion

Treatment outcomes for patients with H1N1 acute respiratory failure improved from 2009 to 2016 in two tertiary referral centers in South Korea.

## Supporting information

S1 Dataset (CSV)
